# Knockdown of NANOG Reduces Cell Proliferation and Induces G0/G1 Cell Cycle Arrest in Human Adipose Stem Cells

**DOI:** 10.3390/ijms20102580

**Published:** 2019-05-26

**Authors:** Maria Pitrone, Giuseppe Pizzolanti, Antonina Coppola, Laura Tomasello, Stefania Martorana, Gianni Pantuso, Carla Giordano

**Affiliations:** 1 Aldo Galluzzo Laboratory of Regenerative Medicine, Department of Health Promotion Sciences, Maternal and infant Care, Internal Medicine and Medical Specialties, PROMISE, University of Palermo, 90127 Palermo, Italy; maria.pitrone@unipa.it (M.P.); antonina.coppola02@unipa.it (A.C.); laura.tomasello@unipa.it (L.T.); 2 ATeN (Advanced Technologies Network Center), University of Palermo, 90127 Palermo, Italy; 3 Department of Surgical, Oncological and Oral Sciences, Division of General and Oncological Surgery, University of Palermo, 90127 Palermo, Italy; stefania.martorana@unipa.it (S.M.); gianni.pantuso@unipa.it (G.P.)

**Keywords:** human adipose stem cell, *NANOG*, cell cycle regulation, *DNMT1*, lentiviral transduction

## Abstract

The core components of regenerative medicine are stem cells with high self-renewal and tissue regeneration potentials. Adult stem cells can be obtained from many organs and tissues. *NANOG*, *SOX2* and *OCT4* represent the core regulatory network that suppresses differentiation-associated genes, maintaining the pluripotency of mesenchymal stem cells. The roles of *NANOG* in maintaining self-renewal and undifferentiated status of adult stem cells are still not perfectly established. In this study we define the effects of downregulation of *NANOG* in maintaining self-renewal and undifferentiated state in mesenchymal stem cells (MSCs) derived from subcutaneous adipose tissue (hASCs). hASCs were expanded and transfected in vitro with short hairpin Lentivirus targeting *NANOG*. Gene suppressions were achieved at both transcript and proteome levels. The effect of *NANOG* knockdown on proliferation after 10 passages and on the cell cycle was evaluated by proliferation assay, colony forming unit (CFU), qRT-PCR and cell cycle analysis by flow-cytometry. Moreover, *NANOG* involvement in differentiation ability was evaluated. We report that downregulation of *NANOG* revealed a decrease in the proliferation and differentiation rate, inducing cell cycle arrest by increasing *p27*/*CDKN1B* (Cyclin-dependent kinase inhibitor 1B) and *p21*/*CDKN1A* (Cyclin-dependent kinase inhibitor 1A) through *p53* and regulate *DLK1*/*PREF1*. Furthermore, *NANOG* induced downregulation of *DNMT1*, a major DNA methyltransferase responsible for maintaining methylation status during DNA replication probably involved in cell cycle regulation. Our study confirms that *NANOG* regulates the complex transcription network of plasticity of the cells, inducing cell cycle arrest and reducing differentiation potential.

## 1. Introduction

*OCT4*, *SOX2* and *NANOG* are the actors in the complex machinery that regulate pluripotency of mesenchymal stem cells [1]. Expression of *OCT4* and *NANOG* is restricted to pluripotent cells, and they are downregulated upon differentiation [2,3]. *OCT4* and *NANOG* work together to maintain pluripotency without LIF (Leukemia inhibitory factor) [4] and studies confirm that *NANOG* downregulation induces ESCs (Embryonic stem cell) differentiation into extraembryonic lineages [5,6]. Wang et al. showed that *OCT4* and *NANOG* maintain self-renewal and block differentiation in ESCs [7]; however, little is known about their mechanism in adult mesenchymal stem cells. Adipose stem cells (ASCs) represent an alternative source of mesenchymal stem cells that can be easily and safely obtained from adipose tissue, growing under appropriate culture conditions for a long time [8,9,10]. As previously shown, ASCs express CD29, CD90, CD105 and CD73 and are negative for the hematopoietic endothelial markers CD34, CD45, CD117, and HLA-DR [11,12,13,14]. Nowadays, it is more important to understand the mechanisms through which maintain the proliferation, differentiation and heterogeneity of these cells [15,16]. *OCT4*, *SOX2* and *NANOG* have also been suggested to play a similar role as embryonic stem cells in adult mesenchymal cells, including human Adipose Stem Cells (hASCs). In our previous studies, we found that the embryonic stem cell marker *NANOG* is over-expressed in MSCs derived from adipose tissue and its silencing with a RNA interference technology causes downregulation of *OCT4* and *SOX2* gene expression [13], confirming recent studies that have demonstrated a central role of *NANOG* in regulating stem cells’ multipotent properties [15,17].

*DLK1* (*PREF1*) is a marker used to characterized mouse preadipocyte progenitors which inhibits adipogenesis, suppressing *C/EBPβ* and *C/EBPδ* gene expression [18,19,20,21,22,23]. Recently, *DLK1* (*PREF1*) proved to be a useful marker for human ASCs [24,25] and methylation regulates its expression. One of the DNA methyltransferase that regulates the DNA methylation during replication is the *DNMT1* [25] and ESCs differentiation and embryo development are regulate by DNA methylation. A recent study [26] demonstrated that *NANOG* directly binds to the promoter of *DNMT1* [26] and enhances its expression. In this study, our aim was to investigate the role of *NANOG* in maintaining the proliferation and differentiation potential of ASCs after downregulation of *NANOG* with a Lentivirus system and to examine the expression of some important genes related to cell growth control such as *p27*/*CDKN1B*, *p21*/*CDKN1A*, *CCDN1* and *PREF1*. We show that *NANOG* downregulation induced a decrease in the proliferation rate and differentiation potential and led to cell cycle arrest in G0/G1 by regulating *CCDN1*, *p21*/*CDKN1A* and *p27*/*CDKN1B* through *p53* and a *PREF1* inhibition, inducing loss of pluripotency via *DNMT1*.

## 2. Results

### 2.1. hASC Isolation and Characterization

**Figure 1:**
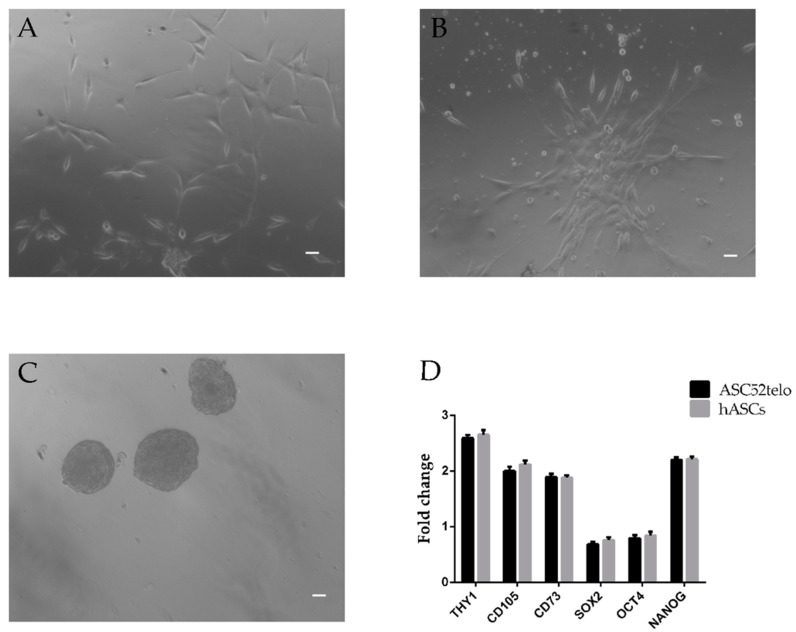
hASC morphology on day 5 (**A**) of expansion culture observed under light microscopy (10×) with phase contrast with a Nikon DS-FI1 CCD camera. Scale bar = 10μm. (**B**) Shows the formation of hASC colonies when cultured in adhesion, Scale bar = 10μm, while (**C**) shows spheres which formed when hASCs were cultured in low adhesion culture conditions, Scale bar = 10μm. A representative experiment referring to 23 samples studied. (**D**) qRT-PCR analysis in freshly isolated hASCs at passage 4 of *THY1*, *CD73*, *CD105*, *OCT4*, *SOX2* and *NANOG* gene expression compared to ASC52telo, hTERT immortalized adipose-derived mesenchymal stem cells. Data shown are relative to an endogenous control (beta-Actin). Relative expression levels were assessed using the 2^−ΔΔ*C*t^ method. Values are shown as the mean ± SE. Data are representative of three independent experiments.

Enzymatic digestion of biopsied human adipose tissue was obtained after consent from 20 patients (nine men and 11 women; age 45 ± 10 years; with body mass index (BMI) range 28 ± 3) undergoing elective open-abdominal surgery. As previously published, hASC highly expressed THY1, CD105 and CD73 and all of the ESC markers SOX-2, OCT4 and NANOG (see Table S1 in Supplementary). When cultured in adhesion we observed the formation of colonies of fibroblastic-like cells, whereas in low-adhesion culture conditions spheres were formed (Figure 1).

### 2.2. Knockdown of NANOG Silencing by Lentivirus

**Figure 2:**
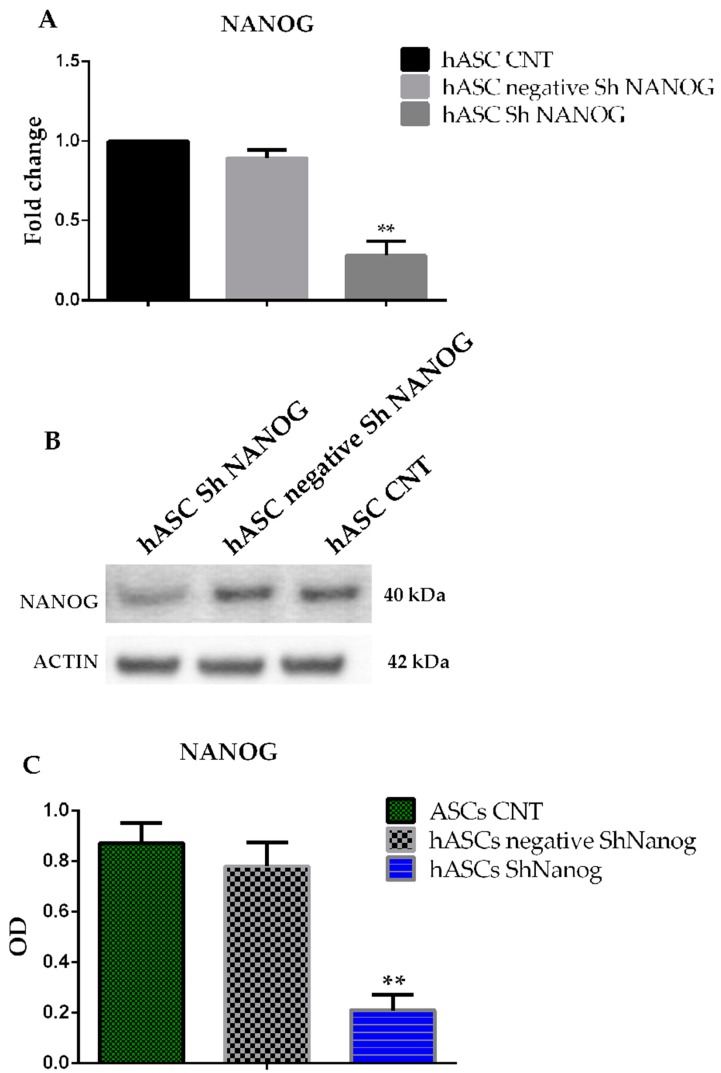
Freshly isolated hASCs at passage 4 (hASCs CNT), hASCs lentivirally transducted with scrambled (negative) or shRNA against *NANOG*. (**A**) qRT-PCR analysis for *NANOG* gene expression after 10-day antibiotic selection. Relative expression levels were assessed using the 2−ΔΔ*C*t method. Data are representative of three independent experiments with the fold change compared to expression levels in a commercial human adipose stem cell line (ASC52telo, hTERT immortalized adipose-derived mesenchymal stem cells). Values are shown as the mean ± SE, ** *p* < 0.01. (**B**,**C**) Western blot analysis for protein expression of NANOG after 15 days of antibiotic selection. Values are shown as mean ± SE, ** *p* < 0.01.

To evaluate the roles of *NANOG* in maintaining stem cell properties we studied changes in stem cell marker gene expression in hASCs lentiviral transducted with shRNA against *NANOG*. The mRNA and protein expression level of NANOG after 10 and 15 days of antibiotic selection after shRNA infection was evaluated by quantitative PCR and western blot analysis. As observed in Figure 2A, the *NANOG* mRNA level decreased by almost 90 ± 3.5% in cell lines infected with *NANOG* shRNA lentivirus as compared to the control group (hASCs CNT, freshly isolated hASCs at passage 4, and hASCs transducted with scrambled shRNA against *NANOG*, hASC negative shRNA *NANOG*). Furthermore, Figure 2B showed the downregulation of NANOG protein expression after 15-day antibiotic selection, by almost 76 ± 2.3% in hASCs infected with *NANOG* shRNA lentivirus (Optical Density (OD) = 0.87 ± 0.9 versus 0.2 ± 0.06). The quantitative analysis of western blot bands was performed using ImageJ. In our previous studies we found that the embryonic stem cell marker NANOG is over-expressed in hASCs and its silencing causes downregulation of the *OCT4* and *SOX2* genes. As assessed by real-time RT-PCR, we confirm that *NANOG* mRNA in freshly hASC is highly expressed and *NANOG* knockdown induces *OCT4* and *SOX2* downregulation, indicating loss of pluripotency in hASCs (data shown in Supplementary Materials).

### 2.3. Analysis of PREF1

**Figure 3:**
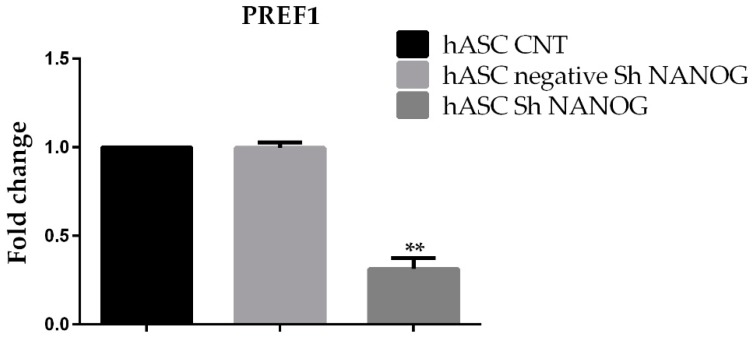
qRT-PCR analysis of *PREF1* gene expression in freshly isolated hASCs at passage 4 (hASCs CNT), hASCs lentivirally transducted with scrambled (negative) and shRNA against *NANOG* (hASCs sh *NANOG*). *PREF1* was significantly downregulated in hASCs sh *NANOG*. Relative expression levels were assessed using the 2^−ΔΔ*C*t^ method. Data are representative of three independent experiments with the fold change compared to expression levels in a commercial human adipose stem cell line (ASC52telo, hTERT immortalized adipose-derived mesenchymal stem cell). The experiment was repeated at least three times ** *p* < 0.01.

In order better to understand the differentiation potential of hASCs biology, we studied the effect of *NANOG* shRNA knockdown on *DLK1*/*PREF1* gene expression. We found that *PREF1* mRNA was downregulated (0.8 ± 0.03 versus 0.3 ± 0.06, *p* < 0.01) in hASCs with *NANOG* downregulated (Figure 3).

### 2.4. Downregulation of NANOG Inhibited Cell Proliferation, Increased Population Doubling Time and Reduced Differentiation Potential

**Figure 4:**
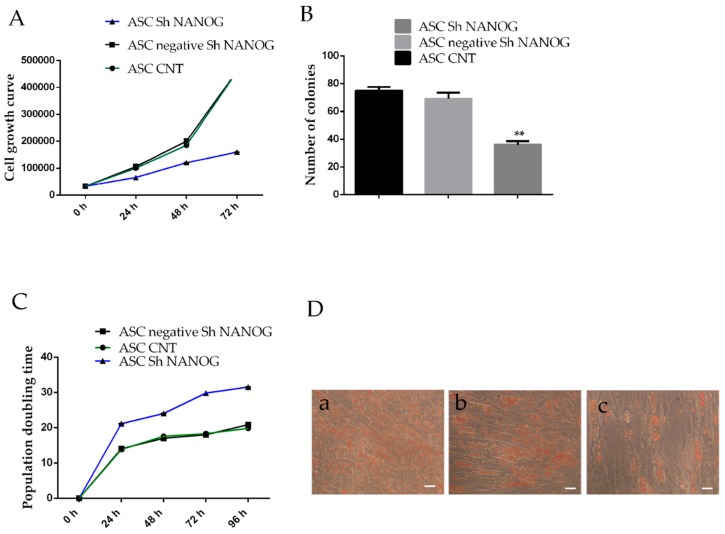
Time-dependent effects of downregulation of *NANOG* on hASCs growth, colony formation ability, population doubling time and differentiation potential. (**A**) Shows the cell growth rate over a 72-h period in freshly isolated hASCs at passage 4 (ASC CNT), hASCs lentivirally transduced with scrambled (negative *NANOG*) or shRNA against NANOG using the trypan blue viability assay. (**B**) The clone numbers (more than 50 cells) of the freshly isolated hASCs at passage 4 (ASC CNT) and hASCs negative Sh *NANOG* were much higher than that of the hASCs sh *NANOG* (75 ± 5 versus 40 ± 5). (**C**) The population doubling time analysis. (**D**) Shows the differentiation potential into adipogenic lineage: (a) hASCs at passage 4 differentiated into adipocytes, (b) hASCs scrambled sh *NANOG* tranfected cells (hASCs negative sh *NANOG*) differentiated into adipocytes and (c) hASCs lentivirally transducted with shRNA against *NANOG* differentiated into adipocytes. Cultures were observed under light microscopy (10×) with phase contrast with a Nikon DS-FI1 CCD camera. scale bar=10μm (Values are mean ± SD, ** *p* < 0.01). A representative photograph of the colonies formed 14 days after lentivirus transfection, stained with crystal violet, is shown in Supplementary Materials.

The effects of *NANOG* knockdown on ASCs proliferation were assessed using trypan blue assay and Colony Forming Unit ability. Cell proliferation analysis shows that the hASCs transducted with shRNA against *NANOG* grew significantly more slowly than wild-type cells and control cells, and after three days, this difference became more evident (Figure 4A). Downregulation of *NANOG* reduces the number of the colonies. As shown in Figure 4B, the clonogenicity of ASCs transfected with shRNA lentivirus *NANOG* decreased, according to the number of cell colonies. The colony formation rate of *NANOG* shRNA-infected cells was 8.5 ± 3.6%, lower than that of freshly isolated hASCs at passage 4 (hASCs CNT) and scrambled sh *NANOG* tranfected cells (hASCs negative sh *NANOG*) (** *p* < 0.01) and the results show an increase in the population doubling time (Figure 4C). ASCs with *NANOG* knockdown also show a decrease in adipogenic differentiation potential (Figure 4D). Taken together, hASCs transducted with shRNA against *NANOG* decrease in proliferation capacity and differentiation potential, indicating that *NANOG* has a role in regulation of stem cell properties.

### 2.5. Knockdown of NANOG Inhibited the Expression of CCND1/Cycline D1, Enhanced the Expression of p21 and p27 and Induced Cell Cycle Block in the G0/G1 Phase

**Figure 5:**
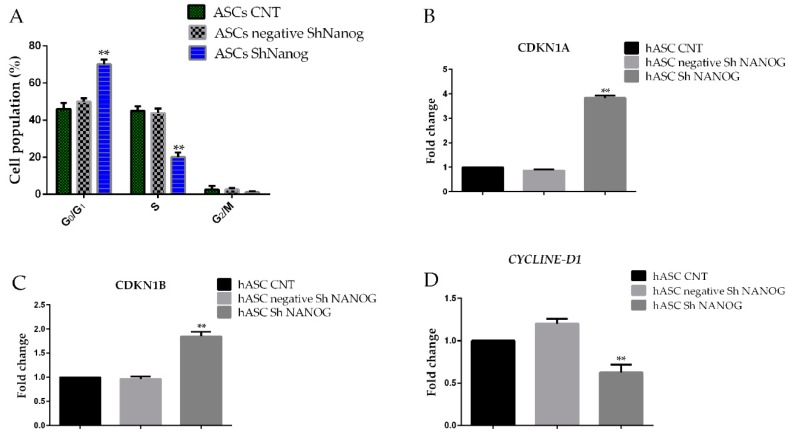
Changes in hASCs cell cycle distribution as a result of *NANOG* downregulation. (**A**) Shows cell cycle distribution of hASCs according to Nicoletti’s protocol. (**B**) and (**C**) Real-time PCR analyses showed that expression of the cell cycle protein CDKN1A and CDKN1B were significantly increased in hASCs with NANOG downregulated while the expression of *CYCLINE D1* was downregulated (**D**). Flow cytometry analyses showed that downregulation of *NANOG* induced a decrease in the proportion of G2/M + S phase cells in hASCs sh *NANOG* as compared to freshly isolated hASCs at passage 4 (hASCs CNT). All experiments were repeated at least three times. (Values are mean ± SD, ** *p* < 0.01).

We evaluated the effects of *NANOG* knockdown on the growth capability of ASCs, and our results confirm that in *NANOG* knockdown the percentage of cells in G0/G1 phase increased from 48 ± 3.2% for freshly isolated hASCs at passage 4 (hASCs CNT) and 52 ± 1.8% for hASCs scrambled shRNA to 70.5 ± 1.2% (*NANOG* knockdown group), while the percentage of S phase cells decreased from 45 ± 2.3% for freshly isolated hASCs at passage 4 and 48% for scrambled (negative shRNA NANOG) to 20% (*NANOG* knockdown group). (Figure 5A) These results indicated that downregulation of NANOG expression arrested hASCs in the G0/G1 phase. Consistent with these observations, downregulation of *NANOG* induced a reduction of the proliferation rate as indicated by the proliferation index (P.I.) 0.52 ± 0.06% in hASCs negative for shRNA against *NANOG* versus P.I 0.23 ± 0.001 in hASCs transfected with shRNA against NANOG. To investigate the G0/G1 phase arrest we evaluated the principal genes involved in cell proliferation, p27/CDKN1B (Cyclin-dependent kinase inhibitor 1B) *p21*/*CDKN1A* (Cyclin-dependent kinase inhibitor 1A) and *CYCLINE D1* by qRT-PCR. Interestingly, as shown in Figure 5, knockdown of *NANOG* in hASCs increased expression of the *CDKN1B* and *CDKN1A* (1.8 ± 0.08-fc and 3.8±0.05-fc, respectively, ** *p* < 0.01) mRNA level when compared to the negative sh *NANOG* and freshly isolated hASCs at passage 4 (hASCs CNT) (0.85 ± 0.05). Significant downexpression of *CYCLINE D1* in mRNA levels was detected (0.63 ± 0.09-fc, ** *p* < 0.01).

### 2.6. Knockdown of NANOG Regulates DNMT1

**Figure 6:**
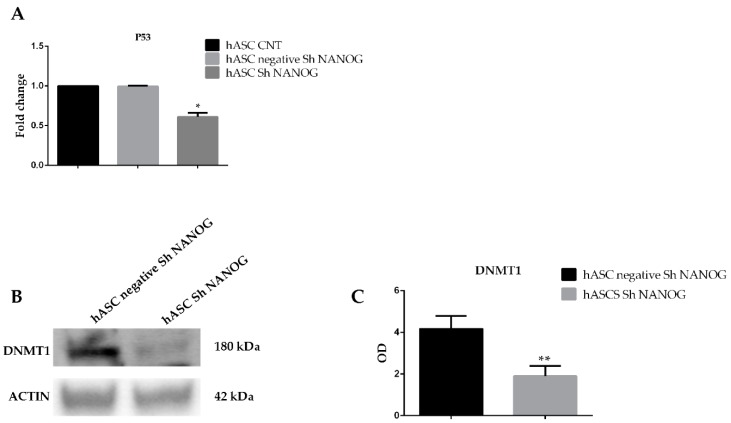
(**A**) qRT-PCR of *p53* gene expression in in freshly isolated hASCs at passage 4 (hASCs CNT), hASCs lentivirally transducted with scrambled (negative) and shRNA against NANOG (hASCs sh NANOG). *p53* gene expression was significantly downregulated following inhibition of NANOG in hASCs. Relative expression levels were assessed using the 2^−ΔΔ*C*t^ method. Data are representative of three independent experiments with the fold change compared to expression levels in a commercial human adipose stem cell line (ASC52telo, hTERT immortalized adipose-derived mesenchymal stem cell). The experiment was repeated at least three times * *p* < 0.05. (**B**) Representative western blot analysis for expression of DNMT1. Densitometric analysis of the Western blot depicted in (**C**). The histograms are the results of three independent experiments. The values are shown as the mean ± SE, ** *p* < 0.01. OD, optical density (** *p* < 0.01).

To clarify the role of p53 in regulation of *p21* and *p27* gene expression in hASCs with *NANOG* downregulated we evaluated p53 mRNA expression. Our findings confirmed that freshly isolated hASCs at passage 4 expressed *p53* and *NANOG* downregulation induced a significant change in *p53* (0.5 ± 0.08-fc * *p* < 0.05) gene expression, as shown in Figure 6A. DNA methylation seems to play an essential role in regulating ESC differentiation and embryo development and *DNMT*-mediated CpG methylation has been demonstrated to play a distinct role in cell cycle regulation. To investigate the possible involvement of *DNMTs* in cell cycle arrest we evaluated expression of the DNMT1 protein in hASCs lentivirally transduced with shRNA targeting *NANOG*. We found that DNMT1 protein levels were dramatically reduced in hASCs with *NANOG* knockdown compared to freshly isolated hASCs at passage 4 and to hASCs scrambled (optical density (OD) = 0.2 ± 0.03 versus 0.4 ± 0.06). (Figure 6B,C).

## 3. Discussion

The properties of stem cells are indicated as the ability to proliferate and to preserve an undifferentiated state and the capacity to differentiate toward multiple cell lineages. It is well known that *SOX2 OCT4* and *NANOG* are the core of “pluripotency” machinery. Our previous study suggested that *NANOG* is an essential transcriptional regulator of genes involved in maintaining undifferentiated pluripotent state of hASCs, but few data exist to conclude that the hASCs fate and differentiation potential are regulated by this protein. In this study hASCs were isolated from biopsies of subcutaneous adipose tissue undergoing elective open-abdominal surgery and were characterized for stem cell markers CD90, CD105 and CD73, and then, cells were transducted with short hairpin Lentivirus system targeting *NANOG*, which resulted in significant downregulation of *NANOG* gene and protein expression. Inhibition of *NANOG* led to a significant downregulation of *OCT4* and *SOX2*, as previously demonstrated [13], and we were able to detect a decrease in expression of the adipose stem cell marker *DLK/PREF1*. Knockdown of *NANOG* in hASCs reduced cell proliferation rate and differentiation potential, inducing cell cycle arrest in G0/G1. p21/CDKN1A and p27/CDKN1B are proteins of the Cip/Kip family that act as key cell cycle regulators by inhibiting CDK [27,28]. To understand the roles of *NANOG* in cell cycle arrest, we examined *p21* and *p27* gene expression. We demonstrate that downregulation of *NANOG* in hASCs induced an increase of *p27* and *p21* gene expression. Recent studies have identified CDK1 as an essential in vivo target of *p27* [29] and our in vitro study confirms that *CCND1* gene expression is reduced. It is well known that *p21* and *p27* expression is regulated largely at the transcriptional level by a *p53*-dependent mechanism [30], which suggests that *NANOG* may regulate expression of these “player” genes through the *p53* tumor suppressor gene. Our experiments confirmed that *NANOG* downregulation induced a reduction of *p53* gene expression, suggesting that *NANOG* can regulate *p21* and *p27* activity through *p53* activity. Furthermore, it is well known that *p21* and *p27* are regulated by methylation [31,32] and DNA methylation regulate the expression of appropriate genes in ESCs [31]. Previous experiments have shown in methylation-deficient mouse embryos (*DNMT1*^−/−^, *DNMT3a*^−/−^ and *DNMT3b*^−/−^) that the restoring of DNA methylation is essential for development [31,32,33]. One of the most important DNA methyltransferases is *DNMT1*, which is responsible for maintaining methylation status during DNA replication, and a recent study demonstrated that *NANOG* directly binds to the promoter of *DNMT1* in ESCs and enhances its expression [26]. To determine whether *NANOG*-mediated maintenance of stem cell properties in hASC came about through regulation of *DNMT1*, we evaluated DNMT1 protein expression. The results confirm that *NANOG* downregulation induced a reduction of DNMT1 protein expression. In conclusion, our results demonstrate that inhibition of *NANOG* not only decreased hASCs growth and induced the arrest of the cell cycle, but also induced a reduction of differentiation ability. Moreover, our results confirm that the stem cell transcription factor *NANOG* regulated cell cycle progression probably via *p53*, which directly controls *p21* and *p27*, key regulator factors of cell cycle machinery. Furthermore, through *DNMT1 NANOG* can methylate *PREF1*, which induces differentiation and can modulate the methylation status of *p21* and *p27* as previously described. All these data suggest that *NANOG* is essential for maintaining hASCs properties.

## 4. Materials and Methods

### 4.1. Cell Culture and Cell Infection with Lentiviral Particles

Subcutaneous (SAT) adipose tissue biopsies were obtained from 23 consenting patients (10 men, 13 women; age 40 ± 10; BMI range between 28 ± 3) undergoing elective open-abdominal and laparoscopy surgery. The protocol was approved by the Independent Ethical Committee (no. 08/2018; 27 August 2018) at the P. Giaccone Azienda Ospedaliera Universitaria Policlinico, Palermo, Italy. All patients gave their written informed consent. Adipose tissue was processed as previously described [13]. Primary cells were used at passage 4 for all experiments. The commercial primary cell line immortalized with a human telomerase reverse transcriptase (ASC52telo, hTERT) (ATCC® SCRC-4000™, American Type Culture Collection, Manassas, VA, USA) was cultured in Mesenchymal Stem Cell Basal Medium (ATCC PCS-500-030).supplemented with Mesenchymal Stem Cell Growth Kit (ATCC PCS-500-040, LGC Standards, Milan, Italy). For lentivirus infection, 2 × 10^4^ hASCs were seeded in six-well plates, and infected with lentivirus (sc-43958-V, Santa Cruz Biotechnology, DBA, Milan, Italy with a the multiplicity of infection (MOI) 10 in the presence of 8 μg/mL polybrene (*sc*-134220, Santa Cruz Biotechnology). At 24 h after infection, the media were removed and replaced with fresh growth media. After 48 h the media were replaced with fresh growth media containing puromycin (1 μg/mL, cod. P9620, Sigma Aldrich, Milan, Italy) to select for infected cells. All experiments were performed 10–15 days after puromycin selection.

### 4.2. RNA Isolation and Quantitative RT-PCR

RNA was isolated in columns from subconfluent cultures of hASC by RNeasy kit (Qiagen, Hamburg, Germany) as previously described [13]. Gene expression was normalized for the housekeeping gene beta-actin (Invitrogen, Milan, Italy). Amplification of specific transcripts was confirmed by melting curve profiles at the end of each PCR. PCR primers *NANOG* (QT01844808), *OCT3/4* (QT00210840), *SOX2* (QT00237601), *THY1* (QT00023569), *CD105* (QT00013335), *CD73* (QT00027279), *p53* (QT00060235), *CCDN1* (QT00495285), *CDKN1A* (QT00062090), *CDKN1B* (QT00998445) were purchased from Qiagen (QuantiTect ® Primer Assays, Qiagen, Hamburg, Germany), *β-Actin* (FORWARD: 5ʹ-GGACTT CGA GCA AGA GAT GG-3ʹ REVERSE: 5ʹ-AGC ACT GTG TTG GCG TAC AG-3ʹ) was purchased from Invitrogen. All reactions were performed using the Quantitect SYBR Green PCR Kit (cod. 204243, Qiagen) on the RotorGene Q Instrument (Qiagen) as previously described. Gene expression of primary cells was compared with a commercial primary cell line immortalized with a human telomerase reverse transcriptase (ASC52telo, hTERT) ATCC® SCRC-4000™, American Type Culture Collection, Manassas, VA, USA), as positive cell controls. Relative expression levels for each gene were assessed using the 2^−ΔΔ*C*t^ method. The results were represented as histograms with GraphPad Prism 6 Software (GraphPad Software, Inc., La Jolla, CA, USA). qRT-PCR analyses for the stem gene were also performed after lentivirus infection experiments.

### 4.3. Western Blot Analysis

Proteins were extracted from adherent cultured cells and separated as previously described [13,34,35]. The antibodies used are as described in Table 1. The secondary antibody was goat anti-mouse IgG-HRP (Amersham, GE Healthcare Europe GmbH, Milan, Italy). Antigen-antibody complexes were visualized using the ECL prime (Amersham, GE Healthcare Europe GmbH, Milan, Italy) on a CCD camera (Chemidoc, Bio-Rad, Milan, Italy). Western blot bands were quantified with ImageJ 1.48 software (National Institutes of Health, Bethesda, MD, USA) and the results were represented as histograms with GraphPad Prism 6 Software (GraphPad Software, Inc., La Jolla, CA, USA).

### 4.4. Analysis of Cell Cycle Status of MSCs with Lentivirus

Single-cell suspensions of control samples and transfected samples were obtained and seeded at a density of 2 × 10^3^ cells/cm^2^ (passage 3), and the DNA content was assessed according to Nicoletti’s protocol [36] as previously described [13]. Data were acquired with CellQuest Pro software (Becton Dickinson, Milan, Italy) and the percentages of G1, S and G2 phase cells were calculated with the MODFIT-LT 5.0 software program (Verity Software House Inc., Topsham, ME, USA).

### 4.5. Colony-Forming Assay

Single-cell suspension ASCs cell lines were seeded in a six-well culture in DMEM/Ham’s F12 1:1 supplemented with 100 unit/mL penicillin, 0.1 mg/mL streptomycin and 10% fetal calf serum (FCS) at a density of 300 cells/well and cultured at 37 °C in 5% CO_2_. After 14 days, the cells were fixed in 4% paraformaldehyde (Sigma Aldrich) and stained with 0.1% crystal violet (Sigma Aldrich). Only the cell groups containing more than 50 cells were considered as colonies. Numbers of colonies were quantified with ImageJ 1.48 software (National Institutes of Health, Bethesda, MD, USA).

### 4.6. Population Doubling and Cell Proliferation Curve

Cell proliferation curve and population doubling time were assessed as previously described [35]. The doubling time (DT) was calculated in accordance with the literature data (http://www.doubling-time.com/compute.php). Three sets of experiments for each sample were used for calculations.

## 5. Conclusions

We examined whether *NANOG* contributes to maintaining cells in an undifferentiated, pluripotent state by activating certain key genes and by silencing others. For this purpose, hASCs were transfected with a lentivirus with shRNA targeting *NANOG* and our results suggest that *NANOG* plays a key role in the hASCs proliferation rate by increasing the expression of *p21* and *p27* and by modulating *PREF1*. In conclusion, we confirm that *NANOG* is an important player in the complex transcription network that regulates pluripotency. We hypothesized that *p21*, *p27* and *PREF1* may be regulated by *DNMT1*, a promotor that is directly bound by *NANOG*, as demonstrated by Tsai et al. [26]. Further experiments are needed to establish the pathway to explain the involvement of *NANOG* in the control of cell cycle progression.

## Figures and Tables

**Table 1:** Primary antibodies used.

Primary Antibody/Localization Marker	Code Number	Diluition	Incubation
NANOG, nuclear and cytoplasmatic	sc-293121, Santa Cruz Biotechnology	1:500	o/n, 4 °C
DNMT1, nuclear	sc-271729, Santa Cruz Biotechnology	1:500	o/n, 4 °C
B-Actin clone AC-74	A5316, Sigma Aldrich	1:10,000	o/n, 4 °C

## Supplementary Materials

Supplementary Materials can be found at https://www.mdpi.com/1422-0067/20/10/2580/s1.

## Abbreviations

MSC	Mesenchymal Stem Cell
hASC	human Adipose Stem Cell
p21/CDKN1A	Cyclin-dependent kinase inhibitor 1A
p27/CDKN1B	Cyclin-dependent kinase inhibitor 1B
