# Supplementary material for: Knockdown of NANOG Reduces Cell Proliferation and Induces G0/G1 Cell Cycle Arrest in Human Adipose Stem Cells
Source: Int J Mol Sci. 2019 May 26;20(10):2580. doi: 10.3390/ijms20102580 (PMC6566573; doi:10.3390/ijms20102580)
Supplement: Supplementary file 1 [file ijms-20-02580-s001.pdf]

Table S1: Calculated fold change values of mesenchymal stem cell genes in hASCs compared to expression levels ASC52telo.

**MSCs**						
	THY 1	CD-105	CD-73	SOX2	OCT4	NANOG
hASC	2.65 ± 0.09	2.12 ± 0.07	1.88 ± 0.05	0.76 ± 0.05	0.84 ± 0.07	2.21 ± 0.05
ASC52telo	2.59 ± 0.056	2.00 ± 0.08	1.89 ± 0.068	0.74 ± 0.064	0.8 ± 0.06	2.195 ± 0.05

Table S2: Calculated fold change values of SOX2 OCT4 and NANOG after NANOG knockdown (** p<0.01)

**MSCs**	**MESENCHYMAL STEM CELL MARKERS**		
	NANOG	SOX 2	OCT4
hASC negative Sh NANOG	0.89 ± 0.05	0.77 ± 0.05	0.81 ± 0.47
hASC Sh NANOG	0.28 ± 0.09	0.32 ± 0.21	0.4 ± 0.33

Figure S1: colony-forming assays showing NANOG downregulation impaired clonogenic potential of hASCs

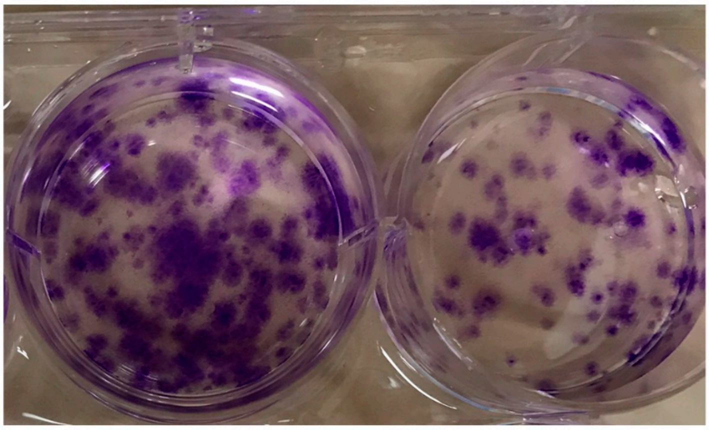

hASC negative Sh NANOG NANOG downregulated

Figure S2: Representative hASC cell cycle distribution as a result of NANOG downregulation

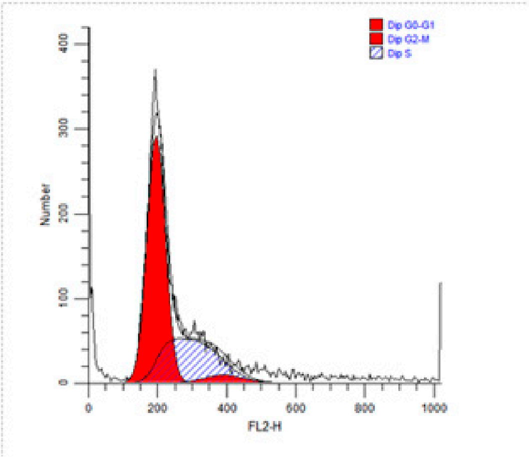

hASC control

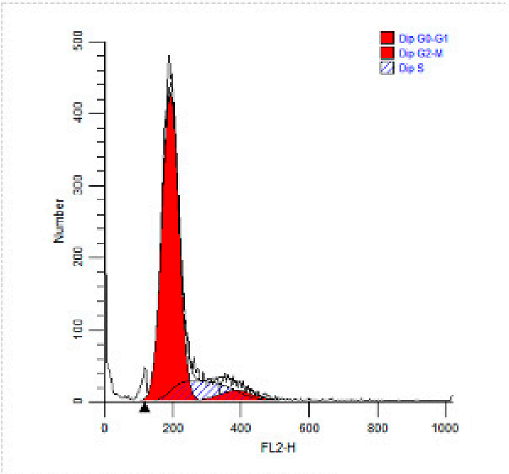

hASC NANOG Knockdown
